# Supplementary figures and images for: Heat Stress Resistance Mechanisms of Two Cucumber Varieties from Different Regions
Source: Int J Mol Sci. 2022 Feb 5;23(3):1817. doi: 10.3390/ijms23031817 (PMC8837171; doi:10.3390/ijms23031817)

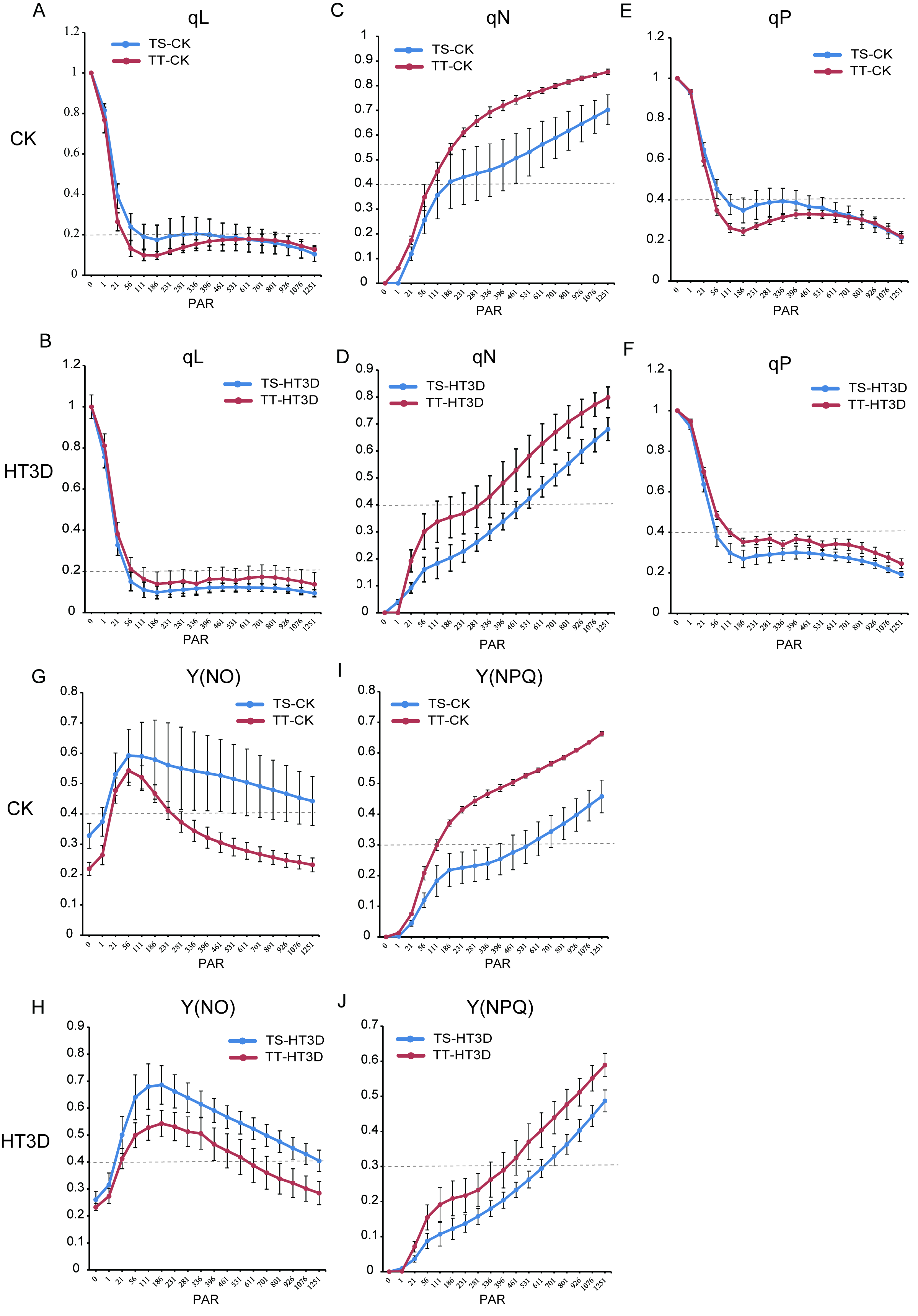

Supplement: Supplementary file 1 [file ijms-23-01817-s001.zip › Supplementary Figure S1.tif]

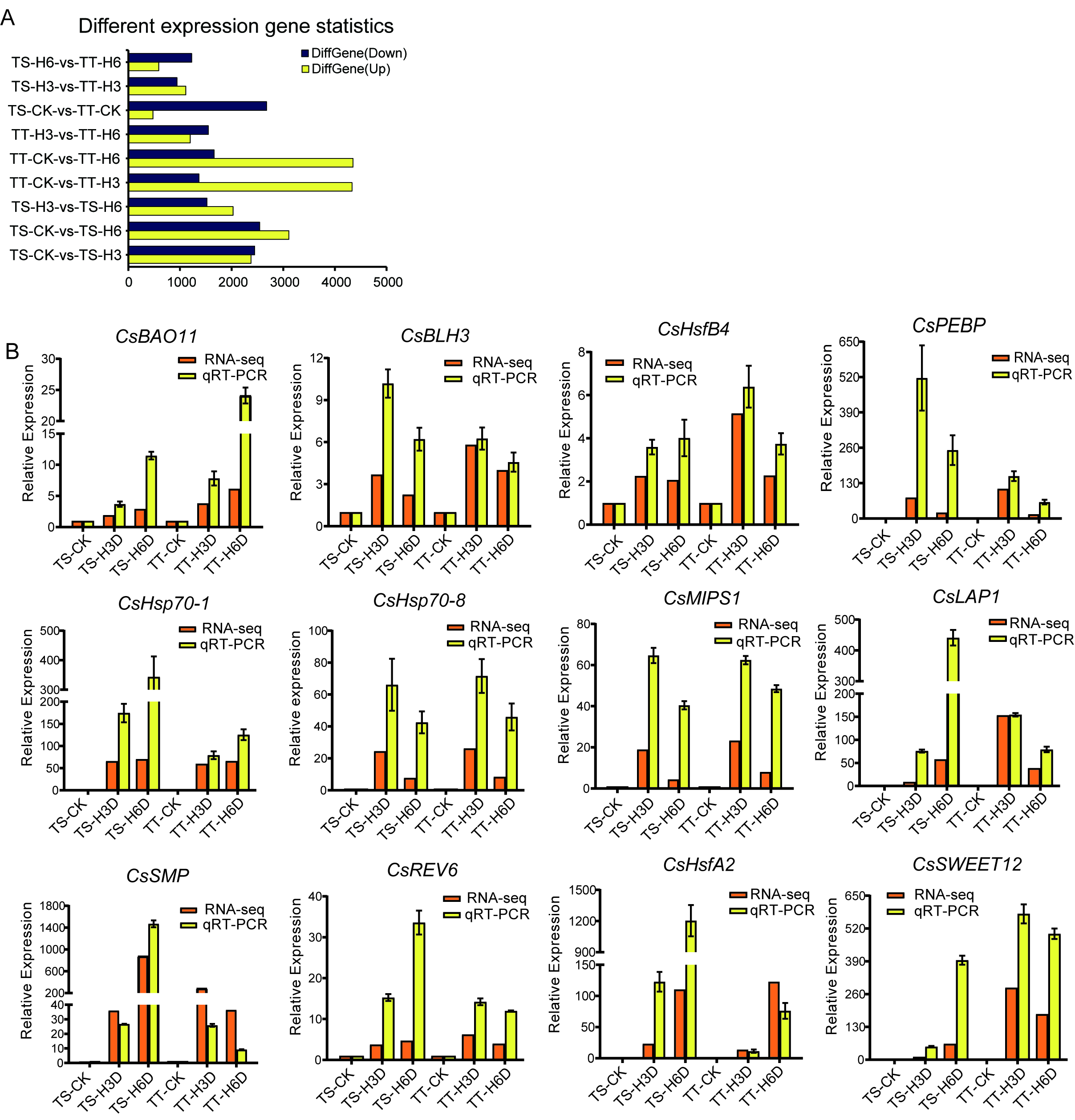

Supplement: Supplementary file 1 [file ijms-23-01817-s001.zip › Supplementary Figure S2.tif]

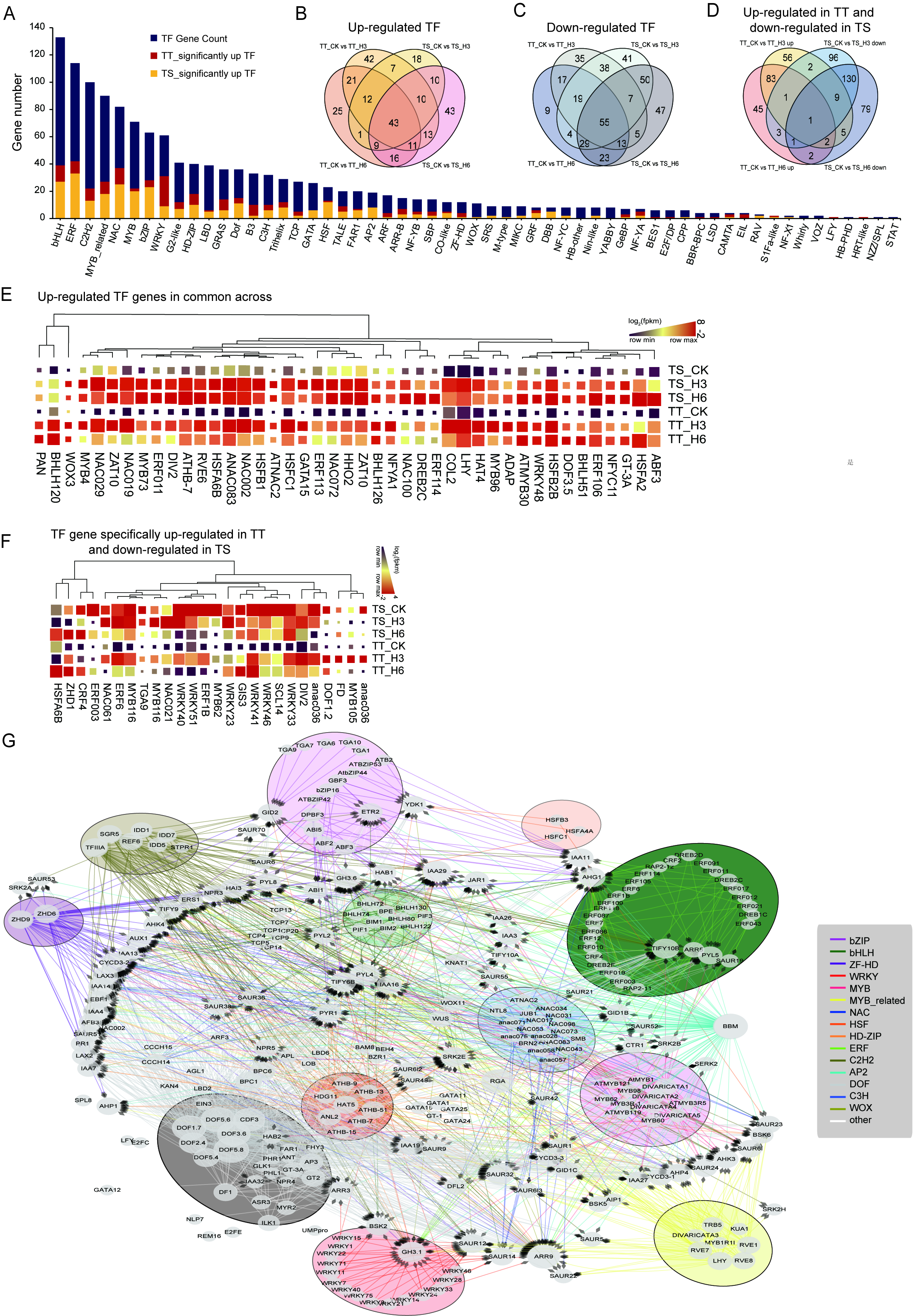

Supplement: Supplementary file 1 [file ijms-23-01817-s001.zip › Supplementary Figure S3.tif]

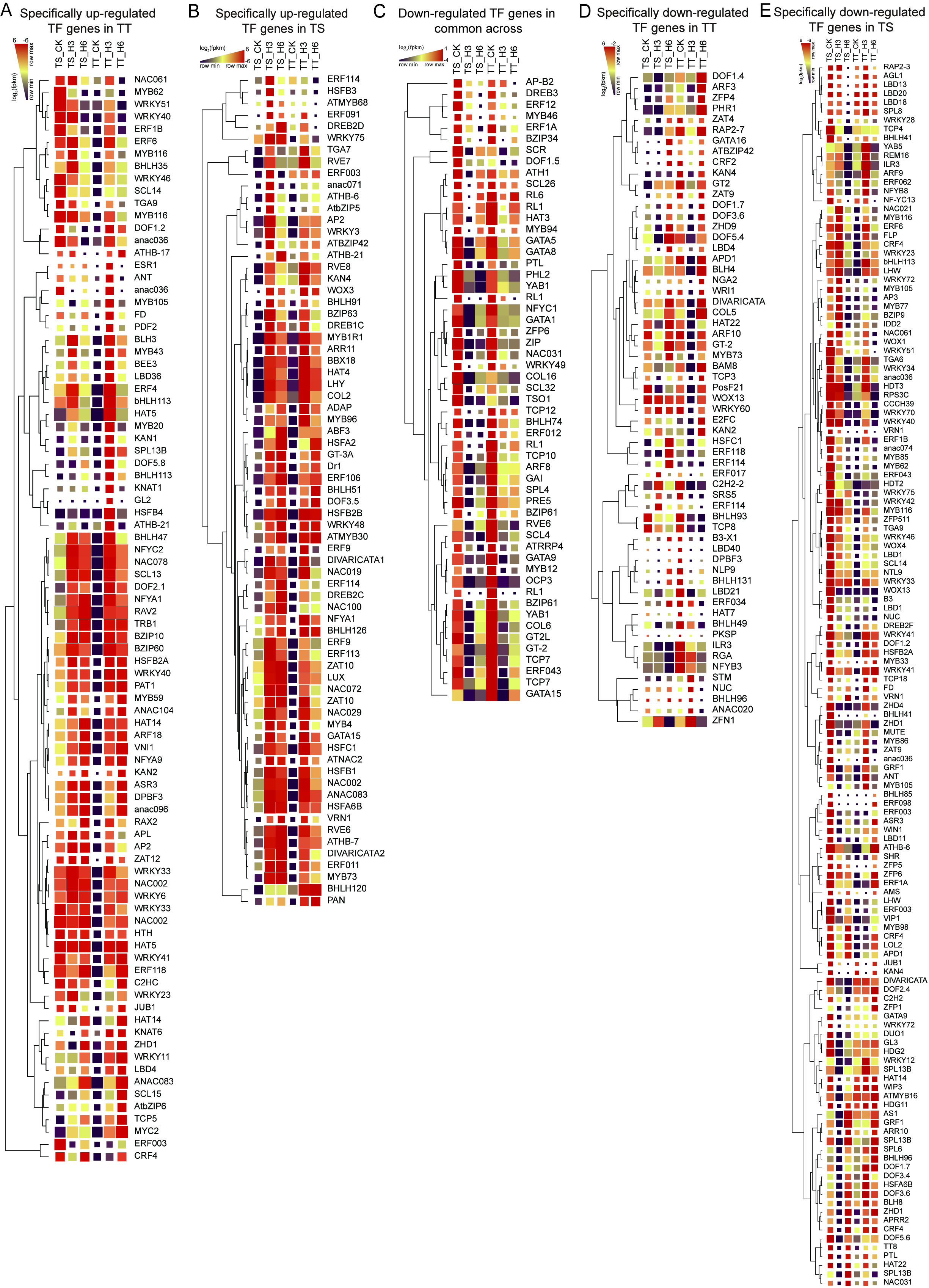

Supplement: Supplementary file 1 [file ijms-23-01817-s001.zip › Supplementary Figure S4.tif]

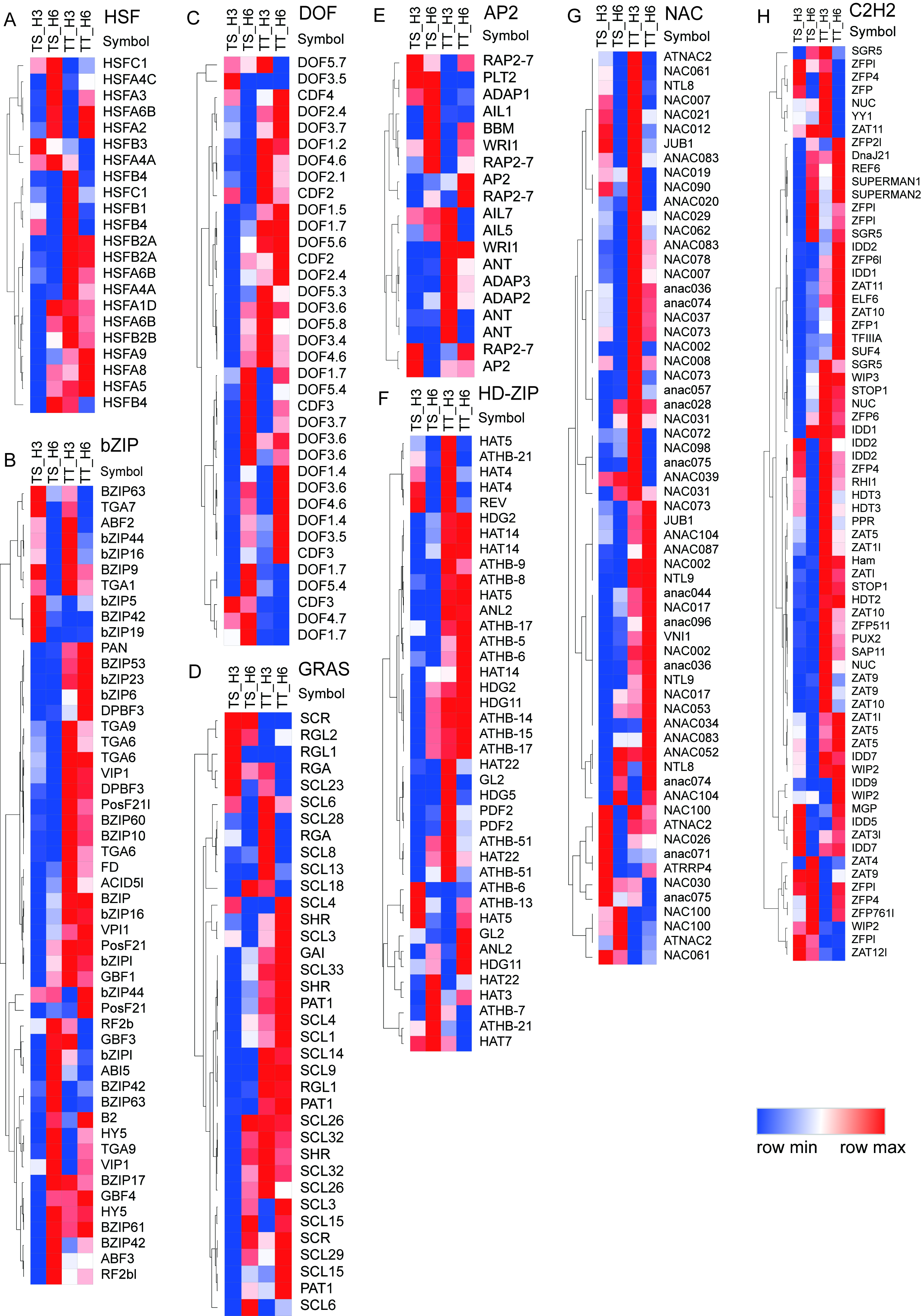

Supplement: Supplementary file 1 [file ijms-23-01817-s001.zip › Supplementary Figure S5-1.tif]

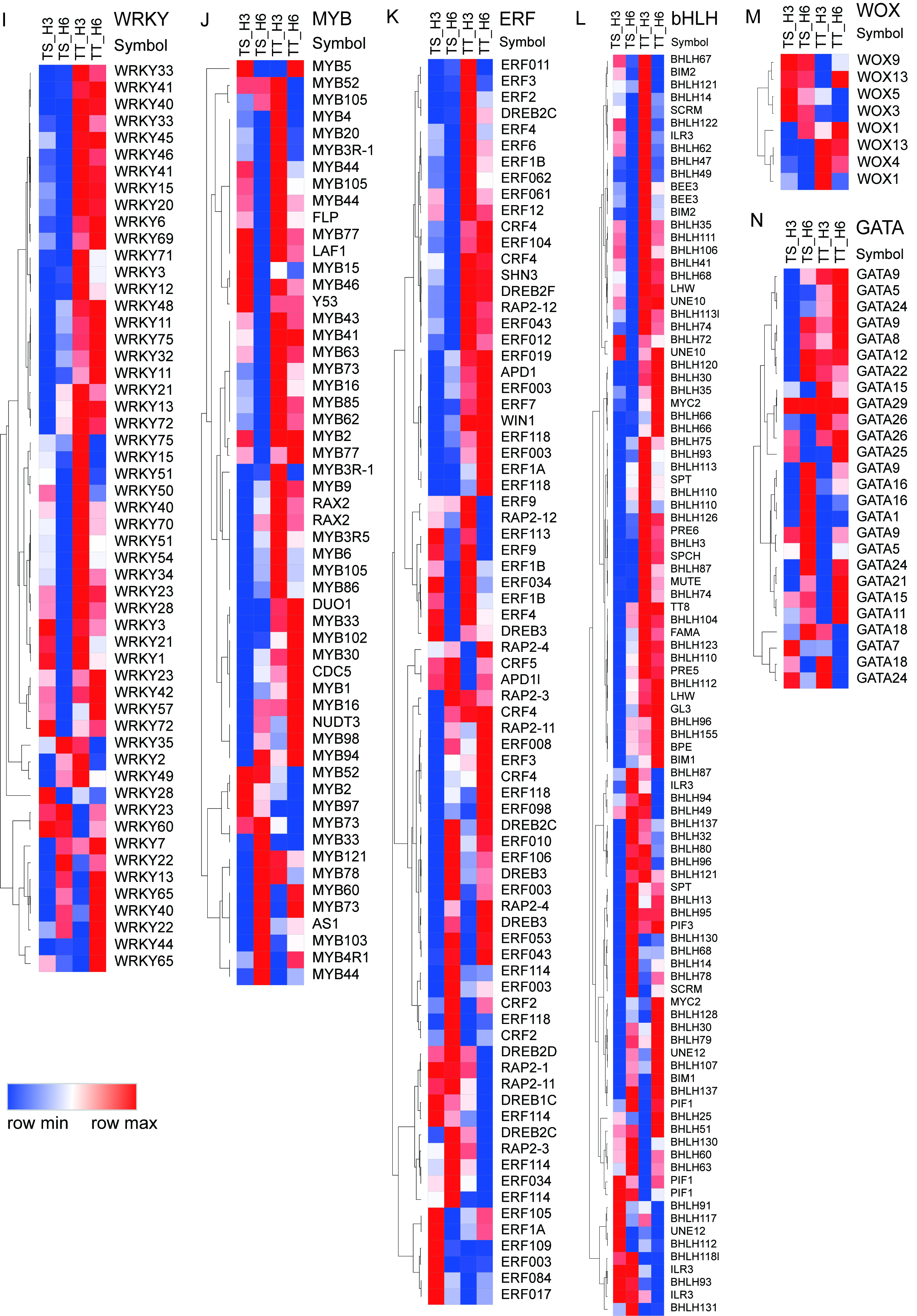

Supplement: Supplementary file 1 [file ijms-23-01817-s001.zip › Supplementary Figure S5-2.tif]
